# Supplementary material for: US workforce gaps in emergency management: A mixed-methods approach of demographics, capacity, and community engagement
Source: PLoS One. 2026 Feb 17;21(2):e0342377. doi: 10.1371/journal.pone.0342377 (PMC12912579; doi:10.1371/journal.pone.0342377)
Supplement: S1 File — (PDF) [file pone.0342377.s001.pdf]

Focus Group Guide:

1. How has the landscape of EM changed?
2. How do you define DEI?
3. What is the current DEI breakdown in your departments? What is the current makeup of your department?
4. Has your office taken steps to improve DEI? Have there been any trainings provided? What areas did the training touch on?
5. How receptive is the community in which you work to outreach provided?
6. What challenges do you face in EM
    a. Has it been challenging to find in position in EM?
    b. What about moving up in EM? What have been the barriers?
7. What have been your sources of support?
8. What do you consider to be the most pressing issue in EM in regards to DEI?
