# Supplementary material for: US workforce gaps in emergency management: A mixed-methods approach of demographics, capacity, and community engagement
Source: PLoS One. 2026 Feb 17;21(2):e0342377. doi: 10.1371/journal.pone.0342377 (PMC12912579; doi:10.1371/journal.pone.0342377)
Supplement: S2 File — (PDF) [file pone.0342377.s002.pdf]

Current Landscape of the Emergency Management Workforce

Start of Block: Introduction

Int This survey is designed to gain a better understanding of the make-up, attitudes, beliefs, and needs of emergency management practitioners across the United States. It is part of a greater research study funded by the Federal Emergency Management Agency (FEMA). It should take approximately 15 minutes to complete. Participants of this survey should be based in the US. Please answer each question honestly, and to the best of your ability. All responses to this survey will remain confidential. Participants can be entered to win one of two $100 gift cards. Thank you for your participation.

End of Block: Introduction

Start of Block: Demographic Section

Q1 What is your gender?

- ○ Male (1)
- ○ Female (2)
- ○ Transgender male (3)
- ○ Transgender female (4)
- ○ Non-binary/third gender (5)
- ○ Prefer not to say (6)

Q2 What is your age?

- 18-24 (1)
- 25-34 (2)
- 35-44 (3)
- 45-54 (4)
- 55-64 (5)
- 65 and above (6)

Q3 What is your race/ethnicity? Please select all that apply:

- American Indian/Alaska Native (1)
- Asian (2)
- Black or African American (3)
- Hispanic or Latino (4)
- Native Hawaiian or Other Pacific Islander (5)
- White (6)

Q4 What is your sexual orientation?

- ◯ Asexual (1)
- ◯ Bisexual (2)
- ◯ Gay (3)
- ◯ Heterosexual or straight (4)
- ◯ Lesbian (5)
- ◯ Pansexual (6)
- ◯ Queer (7)
- ◯ Prefer not to say (8)
- ◯ None of the above, please specify: (9) ____

Q5 Do you consider yourself to have a disability?

- ◯ Yes, I have a physical disability (1)
- ◯ Yes, I have a sensory disability (e.g. visual or hearing impairment) (2)
- ◯ Yes, I have a cognitive or intellectual disability (3)
- ◯ Yes, I have a mental health condition (4)
- ◯ Yes, I have a chronic illness or health condition (5)
- ◯ No, I do not have a disability (6)

Q6 Are you a veteran?

- ◯ Yes (1)
- ◯ No (2)

Q7 What is the highest level of school you have completed or the highest degree you have received?

- ◯ Less than high school degree (1)
- ◯ High school degree or equivalent (e.g. GED) (2)
- ◯ Some college but no degree (3)
- ◯ Associate degree (4)
- ◯ Bachelor degree (5)
- ◯ Graduate degree (6)
- ◯ Doctorate degree (7)

Q8 In what field of study do you have a degree? Select all that apply:

- ▢ Emergency management (1)
- ▢ Homeland security (2)
- ▢ Public Health (3)
- ▢ Other (please specify) (4) ____

Q9 Do you currently have the AEM/CEM credential issued by the International Association of Emergency Managers (IAEM)?

- ○ AEM (1)
- ○ CEM (2)
- ○ No (3)

Q10 Do you have other related credentials or certifications (e.g. ABCP, MBCP, MEP, etc.)?

____

Q11 Which of the following categories best describes your current employment status?

- ○ Student (1)
- ○ Employed (part-time) (2)
- ○ Employed (full-time) (3)
- ○ Not employed, looking for work (4)
- ○ Not employed, not looking for work (5)
- ○ Retired (6)
- ○ Disabled, not able to work (7)

Q12 Approximately how many years have you been working in the field of emergency management:

- ○ 0-2 (1)
- ○ 3-5 (2)
- ○ 6-10 (3)
- ○ 11-15 (4)
- ○ 16-25 (5)
- ○ 26+ (6)

Q13 What is your current job title?

____

Q14 What is your current salary?

- ○ Less than $50,000 (1)
- ○ $50,000-$74,999 (2)
- ○ $75,000-$99,999 (3)
- ○ $100,000-$124,999 (4)
- ○ $125,000-$149,999 (5)
- ○ $150,000-$175,000 (6)
- ○ Greater than $175,000 (7)

Q15 What sector best describes where you work?

- ◯ Consulting (1)
- ◯ Healthcare (2)
- ◯ Higher education (3)
- ◯ K-12 (4)
- ◯ Non-profit (5)
- ◯ Private sector (6)
- ◯ Public sector - County (7)
- ◯ Public sector - Federal (8)
- ◯ Public sector - Local (9)
- ◯ Public sector - State (10)
- ◯ Public sector - Tribal (11)
- ◯ Utilities (12)
- ◯ Other (please specify) (13) ____

Q16 Is emergency management your first career?

- ◯ Yes (1)
- ◯ No (2)

Display this question:

If Is emergency management your first career? = No

Q17 In what field did you previously work?

- ○ EMS (1)
- ○ Fire service (2)
- ○ Law enforcement (3)
- ○ Military (4)
- ○ Public health (5)
- ○ Security (6)
- ○ Social work (7)
- ○ Other (please specify) (8) ____

Q18 In what FEMA region are you located?

◯ Region 1 (Connecticut, Maine, Massachusetts, New Hampshire, Rhode Island, Vermont) (1)

◯ Region 2 (New Jersey, New York, Puerto Rico, Virgin Islands) (2)

◯ Region 3 (Delaware, Maryland, Pennsylvania, Virginia, District of Columbia, West Virginia) (3)

◯ Region 4 (Alabama, Florida, Georgia, Kentucky, Mississippi, North Carolina, South Carolina, Tennessee) (4)

◯ Region 5 (Illinois, Indiana, Michigan, Minnesota, Ohio, Wisconsin) (5)

◯ Region 6 (Arkansas, Louisiana, New Mexico, Oklahoma, Texas) (6)

◯ Region 7 (Iowa, Kansas, Missouri, Nebraska) (7)

◯ Region 8 (Colorado, Montana, North Dakota, South Dakota, Utah, Wyoming) (8)

◯ Region 9 (Arizona, California, Hawaii, Nevada, Guam, American Samoa, Commonwealth of Northern Mariana Islands, Republic of Marshall Islands, Federated States of Micronesia) (9)

◯ Region 10 (Alaska, Idaho, Oregon, Washington) (10)

Q19 Are you fluent in another language besides English?

◯ Yes (1)

◯ No (2)

Display this question:

If Are you fluent in another language besides English? = Yes

Q20 What other language(s) do you speak fluently?

____

Page Break

End of Block: Demographic Section

Start of Block: Organizational Questions

In this section, we will be asking questions about the makeup of your organization as well as your position.

Q21 Which of the following best describes the area in which you work?

- ○ Rural (1)
- ○ Suburban (2)
- ○ Urban (3)
- ○ N/A (4)

Q22 How large is the population that your emergency management office serves?

- ○ < 24,999 (1)
- ○ 25,000-49,999 (2)
- ○ 50,000-74,999 (3)
- ○ 75,000-149,000 (4)
- ○ 150,000-499,999 (5)
- ○ 500,000-1,000,000 (6)
- ○ > 1,000,000 (7)
- ○ N/A (8)

Q23 To whom does your emergency management office report?

- ◯ Environment, Health, & Safety (1)
- ◯ Fire Department (2)
- ◯ Mayor/C-Suite (3)
- ◯ Security (4)
- ◯ Sheriff’s Department/Law enforcement (5)
- ◯ Other (please specify) (6) ____

Q24 Is the head of your emergency management office politically appointed?

- ◯ Yes (1)
- ◯ No (2)
- ◯ I don't know (3)

Q25 How many full-time individuals does your emergency management office employ?

- ○ Less than 1 (it's a part-time or volunteer position) (1)
- ○ 1 (2)
- ○ 2-5 (3)
- ○ 6-10 (4)
- ○ 11-20 (5)
- ○ 21-30 (6)
- ○ 31-50 (7)
- ○ 51-100 (8)
- ○ 100+ (9)

Q26 What have you found to be the most limiting factors in employee retention and promotion within your emergency management career? (rank them in order of importance)

_______ Difficulty balancing work-life commitments (1)
_______ High turnover rate among staff (2)
_______ Inadequate salary and benefits (3)
_______ Insufficient training and professional development programs (4)
_______ Lack of recognition or appreciation for contributions (5)
_______ Lack of or limited opportunities for upward mobility within an organization (6)
_______ Limited opportunities for meaningful work assignments (7)
_______ Poor organizational culture or workplace environment (8)

Q27 On a scale of 1-10 (1 being no impact and 10 being extreme impact), how have funding and resource limitations impacted your emergency management organization?

	0 1 2 3 4 5 6 7 8 9 10
()	

Q28 How strongly impacted is each area by a lack of resources and funding? (1 being no impact and 10 being extremely impacted)

	1	2	3	4	5	6	7	8	9	10
Community preparedness efforts ()										
DEI initiatives ()										
Emergency response capabilities ()										
Workforce expansion ()										
Other (please specify) ()										

Q29 Select the most pressing concerns relating to Diversity, Equity, and Inclusion (DEI) within your organization:

- ☐ Challenges in fostering an inclusive and welcoming work environment for all employees (1)
- ☐ Cultural insensitivity or bias in workplace interacts and practices (2)
- ☐ Disparities in pay and benefits based on identity factors (3)
- ☐ Inadequate resources or support for DEI initiatives (4)
- ☐ Inequitable distribution of workload or assignments (5)
- ☐ Insufficient representation of marginalized groups in decision-making processes (6)
- ☐ Lack of diversity in leadership positions (7)
- ☐ Lack of support from certain individuals or groups toward DEI efforts (8)
- ☐ Limited awareness or understanding of DEI issues among staff (9)
- ☐ Unequal access to career advancement opportunities based on identity factors (10)

Q30 Is there someone within the emergency management office who has DEI (or a related term) in their job title?

- ○ Yes (1)
- ○ No (2)
- ○ Other (please specify) (3) ____

Q31 Is there someone within your emergency management office leading DEI efforts (e.g., a DEI Advisory Council)?

- Yes (1)
- No (2)
- Other (please specify) (3) ____

Q32 Select any trainings you have received through your organization:

- Bystander communication (1)
- Cultural competence (2)
- Cultivating empathy (3)
- Disability awareness (4)
- Implicit bias training (5)
- Inclusive hiring practices (6)
- Intentional inclusion (7)
- Microaggressions (8)
- Sexual harassment prevention (9)
- Other (please specify) (10) ____

Q33 Have you received trainings concerning DEI in your organization?

- Yes (1)
- No (2)
- Other (please specify) (3) ____

Q34 In what ways, if any, do you see your emergency management office incorporating DEI practices? (select all that apply)

- Creating inclusive hiring practices to attract diverse candidates (1)
- Conducting regular diversity audits to assess progress and identify areas for improvement (2)
- Encouraging open dialogue and feedback on DEI initiatives within the office. (3)
- Engaging in community outreach and partnerships with diverse organizations (4)
- Establishing diversity-focused affinity groups or committees (5)
- Implementing diversity training programs for staff (6)
- Incorporating DEI principles into emergency response plans and procedures (7)
- Promoting cultural competency and sensitivity among staff (8)
- Providing resources and support for underrepresented groups within the office (9)
- Reviewing and revising policies to ensure they promote equity and inclusion (10)

Q35 Academic qualifications (Bachelor's or higher) should be required to work in emergency management

- ○ Strongly disagree (1)
- ○ Somewhat disagree (2)
- ○ Neither agree nor disagree (3)
- ○ Somewhat agree (4)
- ○ Strongly agree (5)

Page Break

End of Block: Organizational Questions

Start of Block: Community Questions

In this section, we will be asking questions about your organization's involvement with the community it serves to protect.

Q36 How would you define DEI in emergency management? (rank in order of importance)
______ Equal opportunity for all voices to be heard and represented (1)
______ Forming connections within the community (2)
______ Providing support to people who need it the most (3)
______ Reflecting the community in which you work (4)

Q37 What, if any, have been the most limiting factors in making meaningful steps toward incorporating DEI practices in your community? (select all that apply)

- ▢ Challenges in finding common ground or consensus among diverse community groups. (1)
- ▢ Cultural or linguistic barriers hindering effective communication about DEI topics (2)
- ▢ Fear of change or disruption to existing power dynamics (3)
- ▢ Historical prejudices or biases that impede progress (4)
- ▢ Inadequate representation of diverse voices in decision-making processes (5)
- ▢ Insufficient leadership support or commitment to DEI efforts (6)
- ▢ Lack of funding or resources dedicated to DEI initiatives (7)
- ▢ Limited awareness or understanding of DEI issues within the community (8)
- ▢ Resistance or pushback from community members or stakeholders (9)
- ▢ Structural barriers or systemic inequalities within the community (10)

Q38 Rank these ideas based on how effective they would be in improving community relations and connections:

______ Adding more opportunities for emergency managers to volunteer in communities (1)
______ Offering internship opportunities to involve youth from the community (2)
______ Defining roles within emergency management more clearly (3)
______ Adding mandatory training about cultural awareness (4)
______ Creating organizational standards surrounding DEI (5)
______ Creating a community outreach program to accurately reflect and understand community needs (6)

Q39 Rate how strongly each of the following concerns have impacted community connections: (1 being no impact and 10 being extreme impact)

	1	2	3	4	5	6	7	8	9	10
Lack of transparency with the community ()										
Lack of trust with community leaders ()										
Lack of trust with community members ()										
Language barriers ()										
Physical and technological lack of access to services ()										
Socioeconomic differences ()										
Underrepresentation of minority groups in emergency planning ()										

Q40 Select which statement best applies:

	Strongly disagree (1)	Somewhat disagree (2)	Neither agree nor disagree (3)	Somewhat agree (4)	Strongly agree (5)
I feel connected to the community through my organization (1)	○	○	○	○	○
The community is aware of the work we do (2)	○	○	○	○	○
The community is receptive to the work we do (3)	○	○	○	○	○
I work directly with the community (4)	○	○	○	○	○

Q41 Are you aware of FEMA's Next Generation Core Competencies?

○ Yes (1)

○ No (2)

Display this question:

If Are you aware of FEMA's Next Generation Core Competencies? = Yes

Q42 Have you used them in your role?

- ◯ Yes (1)
- ◯ No (2)

Page Break

End of Block: Community Questions

Start of Block: Conclusion

Q43 Are there additional thoughts or comments you would like to share?

____

Q44 If you would like to be entered into the raffle to receive one of two $100 gift cards, please enter your email address:

____

End of Block: Conclusion
