# Supplementary material for: US workforce gaps in emergency management: A mixed-methods approach of demographics, capacity, and community engagement
Source: PLoS One. 2026 Feb 17;21(2):e0342377. doi: 10.1371/journal.pone.0342377 (PMC12912579; doi:10.1371/journal.pone.0342377)
Supplement: Attachment — Submitted filename: Response to Reviewers.pdf [file pone.0342377.s007.pdf]

Response to Reviewers

Author Response:
We thank the editor and reviewers for their rigorous feedback. We would like to also thank the editor for the opportunity to revise and resubmit the article. We believe that the changes that have been made throughout have made for a stronger submission.

Journal Requirements:
When submitting your revision, we need you to address these additional requirements.

Requirement 1: Please ensure that your manuscript meets PLOS ONE's style requirements, including those for file naming. The PLOS ONE style templates can be found at https://journals.plos.org/plosone/s/file?id=wjVg/PLOSOne_formatting_sample_main_body.pdf and https://journals.plos.org/plosone/s/file?id=ba62/PLOSOne_formatting_sample_title_authors_affiliations.pdf.

Response 1: Thank you for the reminder. We have reviewed our paper to ensure that it meets the journal's style requirements.

Requirement 2: We note that your Data Availability Statement is currently as follows: [All relevant data are within the manuscript and its Supporting Information files.]
Please confirm at this time whether or not your submission contains all raw data required to replicate the results of your study. Authors must share the "minimal data set" for their submission. PLOS defines the minimal data set to consist of the data required to replicate all study findings reported in the article, as well as related metadata and methods (https://journals.plos.org/plosone/s/data-availability#loc-minimal-data-set-definition).
For example, authors should submit the following data:
- The values behind the means, standard deviations and other measures reported;
- The values used to build graphs;
- The points extracted from images for analysis.

Authors do not need to submit their entire data set if only a portion of the data was used in the reported study.
If your submission does not contain these data, please either upload them as Supporting Information files or deposit them to a stable, public repository and provide us with the relevant URLs, DOIs, or accession numbers. For a list of recommended repositories, please see https://journals.plos.org/plosone/s/recommended-repositories.
If there are ethical or legal restrictions on sharing a de-identified data set, please explain them in detail (e.g., data contain potentially sensitive information, data are owned by a third-party organization, etc.) and who has imposed them (e.g., an ethics committee). Please also provide contact information for a data access committee, ethics committee, or other institutional body to which data requests may be sent. If data are owned by a third party, please indicate how others may request data access.

Response 2: Thank you for this comment. We have included all of the aggregated data in our analysis. For some of the responses, the numbers are very low (<5) that risk the identification of the individuals who participated and would compromise their confidentiality. In addition, our institutional review board approval did not include make the dataset available.

Additional Editor Comments:
Comment: Information is provided regarding the topics covered in the focus groups and the survey sections. However, the exact wording of the questions used in both instruments is not fully available in the provided materials, which may limit the precise replication of the study. To improve replicability , the authors are encouraged to consider making the full questionnaires for both the focus groups and the survey available, possibly as supplementary supporting material. These changes are substantial as they directly affect the validity of the conclusions drawn from the survey data and the transparency of the analytical methods employed, in line with PLOS ONE standards. Once these issues are addressed, the manuscript will have a stronger foundation for publication.

Response: Thank you for your feedback.The full focus group and survey questionnaires have been added as supplementary supporting material.

Comment 2: The methodology section mentions the use of logistic regression to identify significant relationships between variables; however, the results of this analysis are not presented anywhere in the Results section or in the tables. This constitutes a notable inconsistency between what is reported in the Methods and what is shown in the Results. The authors should either include the results of the logistic regression or, alternatively, remove the reference to this analysis from the Methods section.

Response: Thank you for your feedback. The reference to this analysis from the Methods section has been removed.

Comment: The authors state that a "nationally representative" survey was conducted. However, in the Discussion section, they mention a low response rate (2%) and that the majority of responses came from only two organizations. These factors significantly limit the generalizability of the survey results and call into question the claim of national representativeness as currently stated. The authors should revise the description of the survey to accurately reflect these limitations, potentially qualifying its representativeness. In addition, the discussion on the implications of this limitation for the generalizability of the survey findings should be expanded and approached with greater caution.

Response: Thank you for this comment. The description of the survey was altered from 'nationally representative survey' to 'nationally disseminated survey' as we believe that more accurately represents the applicability of the survey. As mentioned by the reviewers, the low response rate may compromise the generizability of the survey results, so we also additionally expanded on that in the limitations section in the discussion.

Comment: Results: The results from the focus groups (common themes, selected quotations) and the survey (descriptive statistics presented in tables) are clearly presented and consistent with the descriptive methods employed (thematic analysis for focus groups, frequencies/percentages for the survey). As previously mentioned, while the Methods section describes the use of statistical analyses such as logistic regression, the results of such analyses are not presented in the provided materials.

Recommendation: Based on the evaluation, the article presents valuable information and addresses an important topic using a suitable mixed-methods approach. However, there are significant issues regarding the presentation of the methodology and results, as well as in the interpretation of the survey's representativeness, which affect the quality and validity of certain key claims.

[Note: HTML markup is below. Please do not edit.]

Reviewers' Comments:

Reviewer #1:
Dear authors,
Given the importance of the subject of disasters, the following points can be useful in improving your valuable article.

Comment: The abstract should be properly based on the structure of the journal, including introduction, method, results, and findings.

Response: Thank you for your feedback. The abstract was separated into different sections: Introduction, Method, and Results/Findings. It follows all listed requirements by the journal.

Comment: Keywords should be selected based on the network.

Response: Thank you for your comment. The following keywords have been added: "emergency management, workforce development, capacity building."

Comment: The method should be stated more precisely in the abstract.

Response: Thank you for your feedback. The abstract was revised to include a broader explanation of the method. We incorporated more detail into who the survey was disseminated to, the period of time it was open for, and the incentivization used for the survey.

Comment: The purpose of the research should be properly stated in the abstract and introduction.

Response: Thank you for this comment. We expanded on the purpose in the abstract and in the introduction.

Comment: In the introduction: The topic is not well-explained. The scope of the increase in disasters and their consequences, based on international reports, is not mentioned. Global and American statistics should be referred to, and then the situation of the United States should be examined.

Response: Thank you for this comment. We have now included the scope of the increase in disasters and their consequences using both US and international statistics and references.

Comment: The research gap in the present study is not well explained.

Response: Thank you for this comment. We have added a sentence in the Introduction to better frame the lack of research in this area and the need for our submitted study.

Comment: What are the findings of your study?
The method is not well-explained. How to select people. Criteria and input, and output flow. What is the sample size formula? Questions related to this topic have not been answered. It is suggested that each of the questions in this section about the focus group method be presented, and it is suggested that abbreviations be used in the text. The sample size in this type of study should be mentioned with the source. Why is it this number? The article lacks a source in the method. It should be corrected.

Response: Thank you for this comment. We have clarified how the selection process for the focus group occurred in the Method section, and explained our method further..

Comment: This article does not include a discussion on the topic of the article. Conclusions based on content are not considered a discussion.

Response: Thank you for this important observation. We had included this point in the Introduction and reiterated it again in the Discussion section.

Reviewer #2:

Comment: While Atlas.ti was used for coding, the manuscript does not describe the coding process, development of the codebook, inter-coder reliability, or whether the thematic analysis followed a specific framework.

Response: Thank you for pointing this out. We have added details regarding the coding process in the Methods section.

Comment: The manuscript does not state whether the survey was tested or validated before deployment. Some questions may be susceptible to bias without that validation step.

Response 15: Thank you for this comment. We added information in the Discussion in the limitations section expanding on this point.

Comment: Frequency and logistic regression are briefly mentioned, but no regression results (e.g. odds ratios, confidence intervals, p-values) are reported. This significantly limits interpretability and undermines the credibility of quantitative inferences.

Response: Thank you for this comment. We have removed any reference to regression.

Comment: Expand the Methods section to detail the survey design process (including item construction and pilot testing).

Response Thank you for this comment. We have added additional details regarding the survey design process in the Methods section.

Comment: Report logistic regression outputs or remove the claim of inferential analysis.

Response: Thank you for this comment. We have removed any reference to regression analysis.

Comment: Clarify how qualitative themes were derived and supported with rigor.

Response: Thank you for this point. We have added clarification about the qualitative analysis in the Methods section.

Conclusions are supported by the data and presented appropriately
The conclusions are well-aligned with the data. The manuscript effectively integrates focus group narratives with survey responses to support broader claims about funding challenges, workforce composition, and DEI efforts. In particular, the discussion is balanced in acknowledging strengths (e.g., improving gender representation) and weaknesses (e.g., persistent racial homogeneity) within the EM workforce.

Comment: However, some findings, such as improving gender diversity, could benefit from more nuance, especially given the modest sample sizes and potential non-response bias.

Response: Thank you for this thoughtful feedback. We agree and have revised the Discussion to add nuance around the findings on gender diversity, noting the modest sample size and potential non-response bias. We now frame these findings as preliminary observations rather than definitive trends.

The article is intelligible and written in standard English
The manuscript is mostly well-written and accessible. Nevertheless, several minor grammatical errors and repetitive sentence structures (e.g., "age" repeated twice in one sentence; inconsistent verb tenses) detract slightly from readability.

Comment: A thorough copyediting pass is recommended to improve sentence flow and technical precision.

Response: Thank you for this comment. We have gone through the manuscript to refine the flow and technical precision.

Additional Comments and Recommendations
Comment: Low Response Rate (2%): The manuscript should provide a stronger justification for this low survey response rate and discuss the potential for selection bias. For example, overrepresentation from a few organizations may skew results toward specific perspectives.

Response: Thank you for raising this important point. We acknowledge that the survey yielded a low response rate (2%), which limits the generalizability of the findings. This is a recognized challenge in surveys targeting emergency management professionals, who often have demanding schedules and limited availability for voluntary research participation.

Several factors may have contributed to the low response, including competing responsibilities, survey fatigue, and limitations in the dissemination method. While we cannot eliminate the possibility of response bias, for example, that those more engaged with or interested in the topic may have been more likely to respond, we believe the responses provide valuable insight into the current state of emergency management.

We have expanded the limitations section to reflect these concerns and to clarify that the findings are best viewed as exploratory and hypothesis-generating. Despite the low response rate, the data offer a starting point for future research and help to illuminate areas for further inquiry and policy development.

Comment: Sample Representativeness: The study claims to use a 'nationally representative' survey, but does not describe how representativeness was ensured. Consider adjusting this claim or providing a clearer sampling strategy and weighting information.

Response: Thank you for this comment. We have revised the claim of ‘nationally representative’ to more accurately describe the survey.

Comment: Clarity on Inclusion/Exclusion Criteria: Neither the qualitative nor quantitative sections provide details on participant eligibility criteria beyond membership in EM organizations. This should be explicitly stated.

Response: Thank you for this comment. We have added additional details regarding eligibility criteria in the Methods section.

Comment: Consideration of Intersectionality: While demographic breakdowns are extensive, analysis could be enriched by addressing intersectional experiences, for instance, how gender and race/ethnicity together shape experiences in EM.

Response: Thank you for this comment. We have conducted additional stratified analyses and briefly discuss the main findings in the Discussion section.

Comment: Future Directions Section: This is well developed and proposes compelling avenues for further work, such as mental health in EM and standardization of credentials. The authors may also consider suggesting policy implications from their findings.

Response: Thank you for this comment. We have added a short section in the Discussion section to address this important point.

Comment: The manuscript offers important contributions to the literature on workforce development, equity, and operational challenges in EM. Although the study is well-conceived and its conclusions are largely supported by the data, several methodological and reporting issues must be addressed before the manuscript can be recommended for publication.

Response: Thank you for this point. We have addressed the methodological and reporting issues that the reviewers have pointed out throughout the manuscript and feel that it has greatly strengthened the paper.
