# Supplementary material for: US workforce gaps in emergency management: A mixed-methods approach of demographics, capacity, and community engagement
Source: PLoS One. 2026 Feb 17;21(2):e0342377. doi: 10.1371/journal.pone.0342377 (PMC12912579; doi:10.1371/journal.pone.0342377)
Supplement: Attachment — Submitted filename: renamed_25b7a.docx [file pone.0342377.s006.docx]

**Revision of ‘PONE-D-25-17329_R1’**

**General Assessment**

The manuscript explores the composition and challenges of the emergency management workforce using a mixed-methods design. The topic is relevant and timely, and the study aims to fill a clear gap in understanding workforce diversity and organizational issues within this field. However, while the paper presents descriptive results with clarity, the methodological integration between qualitative and quantitative components is weak, and the qualitative analysis lacks depth and transparency. The manuscript would benefit from a more rigorous approach to mixed-methods reporting, as well as greater methodological detail and interpretive caution in its conclusions. Therefore, I recommend major revision before the manuscript can be considered suitable for publication.

**Originality and Relevance**

The study presents original research not previously published elsewhere, and its focus on workforce composition, diversity, and retention in emergency management contributes to an underexplored area. However, the originality is somewhat limited by the descriptive nature of both data sets and by the absence of strong analytical connections between them. The paper would gain impact if it moved beyond summary reporting to interpretive synthesis, linking qualitative findings to survey patterns or using focus group insights to explain observed trends.

**Technical and Analytical Standards**

The quantitative part of the study is straightforward and replicable. Descriptive statistics are adequate and the tables are clear. The paper should acknowledge that the quantitative findings remain exploratory and descriptive rather than inferential.

The qualitative component raises more serious concerns. The methods section states that Atlas.ti was used for coding, but it does not describe how the codebook was developed, whether coding was iterative, or whether inter-coder reliability was established. The absence of references to analytical frameworks (for example, Braun and Clarke’s thematic analysis or grounded theory procedures) weakens the credibility of the thematic synthesis. Selected quotes are presented, but they serve as illustrations rather than evidence of systematic interpretation. The analysis reads as a set of themes compiled from responses rather than the outcome of rigorous qualitative inquiry.

Given that this is a mixed-methods study, a clear explanation of how qualitative and quantitative data informed each other is missing. The paper describes the focus groups as informing the survey design, but there is no evidence of integration in the analysis or discussion phases. To meet mixed-methods standards, the authors should explicitly describe the rationale for combining methods, the point of integration, and how qualitative insights complement or explain survey findings. For a better understanding of the required integration of quantitative and qualitative data, I would suggest reading this article: https://www.sciencedirect.com/science/article/pii/S0959475225001367

**Conclusions and Support by Data**

The conclusions are generally consistent with the data but sometimes overstate the strength and generalizability of the results. The claim that the study describes the “current landscape” of emergency management in the United States is overstated given the 2 % survey response rate and the concentration of responses within two organizations. The discussion appropriately notes this limitation, yet some interpretive statements (for example, regarding progress in gender diversity) still read as definitive rather than tentative. The authors should reframe these claims as preliminary observations drawn from a small and potentially biased sample.

**Clarity and Writing Quality**

The manuscript is intelligible and written in standard English, though the prose would benefit from professional copyediting to correct minor redundancies and tense inconsistencies. The organization of sections is logical, and tables are well formatted. However, transitions between qualitative and quantitative results could be smoother, emphasizing how each contributes to the overall argument.

**Ethical and Reporting Standards**

The study received IRB approval and follows ethical standards for research involving human participants. The authors acknowledge restrictions on data sharing due to confidentiality risks, which is acceptable if justified to the journal. However, PLOS ONE requires that minimal data sufficient for replication be made available. The authors should verify that aggregated or anonymized data supporting each table are included as supplementary material.

**Data Availability and Transparency**

The data availability statement indicates that full raw data cannot be shared. Given PLOS ONE’s policy, the authors must ensure that the minimal dataset underlying all findings, including numeric values behind summary statistics, is accessible. The qualitative component would also benefit from transparent reporting of coding materials or at least examples of the codebook and analytic memos, which could be shared as supplementary information without breaching confidentiality.
