# Supplementary material for: US workforce gaps in emergency management: A mixed-methods approach of demographics, capacity, and community engagement
Source: PLoS One. 2026 Feb 17;21(2):e0342377. doi: 10.1371/journal.pone.0342377 (PMC12912579; doi:10.1371/journal.pone.0342377)
Supplement: Attachment — Submitted filename: Response to Reviewers - Oct_Nov Revisions.docx [file pone.0342377.s009.docx]

Response to Reviewers

**Comments to the Author**

2. Is the manuscript technically sound, and do the data support the conclusions?

The manuscript must describe a technically sound piece of scientific research with data that supports the conclusions. Experiments must have been conducted rigorously, with appropriate controls, replication, and sample sizes. The conclusions must be drawn appropriately based on the data presented.

Reviewer #1: No

Reviewer #2: Partly

3. Has the statistical analysis been performed appropriately and rigorously?

Reviewer #1: No

Reviewer #2: Yes

4. Have the authors made all data underlying the findings in their manuscript fully available?

The [PLOS Data policy](https://urldefense.com/v3/__http://track.editorialmanager.com/CL0/http:*2F*2Fwww.plosone.org*2Fstatic*2Fpolicies.action*23sharing/1/010f0199e251ae34-b7022345-450d-483f-8897-bf5a8f4b2880-000000/yT--9VoreCmz9XR7T3SFDxCCsWg6dPdWIefZI3_6WOA=232__;JSUlJSU!!JkGBRS3n8cDS!jDDeNQ_QpHJdvx9IbHhVWtDc_p6sjvt4C5ZAodX0nL5tKXJMWbS0BAgCAtQyVAd6r8MfV7tBXPBcKDmjHr-O$) requires authors to make all data underlying the findings described in their manuscript fully available without restriction, with rare exception (please refer to the Data Availability Statement in the manuscript PDF file). The data should be provided as part of the manuscript or its supporting information, or deposited to a public repository. For example, in addition to summary statistics, the data points behind means, medians and variance measures should be available. If there are restrictions on publicly sharing data—e.g. participant privacy or use of data from a third party—those must be specified.

Reviewer #1: Yes

Reviewer #2: No

5. Is the manuscript presented in an intelligible fashion and written in standard English?

PLOS ONE does not copyedit accepted manuscripts, so the language in submitted articles must be clear, correct, and unambiguous. Any typographical or grammatical errors should be corrected at revision, so please note any specific errors here.

Reviewer #1: Yes

Reviewer #2: Yes

6. Review Comments to the Author

Please use the space provided to explain your answers to the questions above. You may also include additional comments for the author, including concerns about dual publication, research ethics, or publication ethics. (Please upload your review as an attachment if it exceeds 20,000 characters)

**Reviewer #1:**

Dear Author,
Thank you for your efforts, please review the following again.

**Comment:** Abstract: There is no conclusion in this study.

***Response:* Thank you for this comment. We have added a concluding paragraph at the end of the abstract.**

**Comment:** In the keywords, surge capacity is a more accurate option in responding to crises.

***Response:* Thank you for this comment. We have added “surge capacity” to the key words.**

**Comment:** In the introduction, the willingness of the authorities to provide in 2024 and 2025 is presented. In this study, the role of gender is discussed more. If this study is about the role of human resources and the role of its management in crises, this introduction should focus on the pre-hospital topic. This can also affect the title of this study.

***Response:* Thank you for this comment. We have revised the introduction to ensure that the focus of the study is clear.**

**Comment:** In the introduction, the focus of the topic is not well explained.

***Response:* Thank you for bringing this to our attention. We have added several sentences to better focus the introduction.**

**Comment:** Studies conducted show that there is no precise classification in the method. This article can provide a better presentation in a classification method.

***Response:* Thank you for this comment.**

**Comment:** The discussion in this study is not well explained, and the changes are not made clearly.

***Response:* Thank you. We have revised and edited the discussion for clarity.**

**Reviewer #2:** SEE DOCUMENT ATTACHED

The manuscript explores the composition and challenges of the emergency management workforce using a mixed-methods design. The topic is relevant and timely, and the study aims to fill a clear gap in understanding workforce diversity and organizational issues within this field.

**Comment:** However, while the paper presents descriptive results with clarity, the methodological integration between qualitative and quantitative components is weak, and the qualitative analysis lacks depth and transparency. The manuscript would benefit from a more rigorous approach to mixed-methods reporting, as well as greater methodological detail and interpretive caution in its conclusions. Therefore, I recommend major revision before the manuscript can be considered suitable for publication.

***Response:* Thank you for bringing this to our attention. We have added detail to the Methods section to better explain how the focus groups informed the development of the survey. We also reanalyzed our qualitative transcripts to add depth to the results.**

Originality and Relevance:

The study presents original research not previously published elsewhere, and its focus on workforce composition, diversity, and retention in emergency management contributes to an underexplored area.

**Comment:** However, the originality is somewhat limited by the descriptive nature of both data sets and by the absence of strong analytical connections between them. The paper would gain impact if it moved beyond summary reporting to interpretive synthesis, linking qualitative findings to survey patterns or using focus group insights to explain observed trends.

***Response:* Thank you for this helpful comment. We have added a section in the Results titled "Integrating Qualitative and Quantitative Findings” to facilitate the synthesis of the two types of results.**

Technical and Analytical Standards:

The quantitative part of the study is straightforward and replicable. Descriptive statistics are adequate and the tables are clear.

**Comment:** The paper should acknowledge that the quantitative findings remain exploratory and descriptive rather than inferential.

***Response:* Thank you. We have added a sentence in the Discussion that acknowledges this point.**

**Comment:** The qualitative component raises more serious concerns. The methods section states that Atlas.ti was used for coding, but it does not describe how the codebook was developed, whether coding was iterative, or whether inter-coder reliability was established. The absence of references to analytical frameworks (for example, Braun and Clarke’s thematic analysis or grounded theory procedures) weakens the credibility of the thematic synthesis. Selected quotes are presented, but they serve as illustrations rather than evidence of systematic interpretation. The analysis reads as a set of themes compiled from responses rather than the outcome of rigorous qualitative inquiry.

***Response:* Thank you for this important comment. We agree that additional details are necessary to describe the qualitative methods and analysis and have provided them in the methods section. We have also revised the Results section to reflect a more rigorous analytical approach.**

**Comment:** Given that this is a mixed-methods study, a clear explanation of how qualitative and quantitative data informed each other is missing. The paper describes the focus groups as informing the survey design, but there is no evidence of integration in the analysis or discussion phases. To meet mixed-methods standards, the authors should explicitly describe the rationale for combining methods, the point of integration, and how qualitative insights complement or explain survey findings. For a better understanding of the required integration of quantitative and qualitative data, I would suggest reading this article: https://www.sciencedirect.com/science/article/pii/S0959475225001367

**Response: Thank you for this point and for the article. We have added a more detailed explanation to the methods section to explain how the docs group helped inform the development of the survey.**

Conclusions and Support by Data:

The conclusions are generally consistent with the data but sometimes overstate the strength and generalizability of the results.

**Comment:** The claim that the study describes the “current landscape” of emergency management in the United States is overstated given the 2 % survey response rate and the concentration of responses within two organizations. The discussion appropriately notes this limitation, yet some interpretive statements (for example, regarding progress in gender diversity) still read as definitive rather than tentative. The authors should reframe these claims as preliminary observations drawn from a small and potentially biased sample.

**Response: Thank you for this. We have adjusted the title accordingly.**

Clarity and Writing Quality:

**Comment:** The manuscript is intelligible and written in standard English, though the prose would benefit from professional copyediting to correct minor redundancies and tense inconsistencies.

***Response:* Thank you for this comment. We have reviewed the prose and conducted an intensive copyediting to ensure consistency and removal of any redundancy.**

**Comment:** The organization of sections is logical, and tables are well formatted. However, transitions between qualitative and quantitative results could be smoother, emphasizing how each contributes to the overall argument.

***Response:* Thank you for this comment. We have added transitions between qualitative and quantitative data and added a section that discusses the synergy between the two sets of data.**

Ethical and Reporting Standards:

The study received IRB approval and follows ethical standards for research involving human participants. The authors acknowledge restrictions on data sharing due to confidentiality risks, which is acceptable if justified to the journal.

**Comment:** However, PLOS ONE requires that minimal data sufficient for replication be made available. The authors should verify that aggregated or anonymized data supporting each table are included as supplementary material.

***Response:* Thank you for the reminder. We have submitted an IRB amendment to allow for the sharing of aggregated data and will be happy to provide it upon acceptance of the manuscript.**

Data Availability and Transparency:

**Comment:** The data availability statement indicates that full raw data cannot be shared. Given PLOS ONE’s policy, the authors must ensure that the minimal dataset underlying all findings, including numeric values behind summary statistics, is accessible.

**Response: Thank you for this comment. We have confirmed with our IRB office that we share the aggregated data and will be happy to provide it upon acceptance of the manuscript.**

**Comment:** The qualitative component would also benefit from transparent reporting of coding materials or at least examples of the codebook and analytic memos, which could be shared as supplementary information without breaching confidentiality.

***Response:* Thank you for this comment. We have provided the codebook as supplementary information.**
