# Supplementary material for: US workforce gaps in emergency management: A mixed-methods approach of demographics, capacity, and community engagement
Source: PLoS One. 2026 Feb 17;21(2):e0342377. doi: 10.1371/journal.pone.0342377 (PMC12912579; doi:10.1371/journal.pone.0342377)
Supplement: Attachment — Submitted filename: Revision of PONE-D-25-17329R2.docx [file pone.0342377.s008.docx]

**Revision of PONE-D-25-17329R2**

The authors have comprehensively addressed the methodological, presentation, and reporting concerns raised by the reviewers. The manuscript presents an important mixed-methods study, rigorously analyzed in its revised form, and adheres to ethical and data availability standards. The incorporation of a dedicated synthesis section significantly enhances the value of the mixed-methods design.

**Criterion 1. The study presents the results of original research**

The research presented is original and addresses an under-explored area within emergency management. The authors note that existing studies have primarily examined the capacity of state, local, tribal, and territorial (SLTT) governments, but to date, there has been no comprehensive study examining the current workforce landscape across all sectors, including government (SLTT), private, and nonprofit organizations, nor across geographic distinctions (urban, suburban, and rural).

**Criterion 2. Results reported have not been published elsewhere**

The authors affirm that the study presents original research that has not been published previously.

**Criterion 3. Experiments, statistics, and analyses are performed to a high standard and are described in sufficient detail**

The revisions successfully address initial concerns regarding the methodological integration and qualitative analysis rigor. The authors revised the Methods section to provide additional details, specifically explaining how the five focus group sessions informed the development of the large-scale survey. For the qualitative component, the authors confirmed the use of a grounded theory approach and specified the rigorous procedures followed, including the systematic development and documentation of the coding criteria using ATLAS.ti. They also established inter-coder reliability and validity by reviewing twenty percent of the primary coder’s cases, with discrepancies reconciled. Furthermore, the manuscript now includes a dedicated section titled "Integrating Qualitative and Quantitative Findings" to facilitate the interpretive synthesis required in a mixed-methods study.

**Criterion 4. Conclusions are supported by the data and presented appropriately**

The conclusions are well-supported by the synthesis of the mixed-methods data, which showed high levels of concern regarding funding limitations, lack of standardization, risk of burnout, and underrepresentation of minority groups. The authors addressed the initial overstatement of generalizability by adjusting the title and ensuring that interpretive statements are reframed as preliminary observations, considering the low overall survey response rate of 2% and the concentration of responses from two organizations. The revised abstract now includes a concluding paragraph summarizing the key challenges and emerging encouraging trends, such as greater gender diversity and participation from younger professionals.

**Criterion 5. The article is intelligible and written in standard English**

The manuscript is intelligible and written in standard English. Following the prior recommendation for copyediting, the authors reviewed the prose and conducted intensive copyediting to address minor redundancies and ensure consistency. They also improved the organization by adding transitions and a section dedicated to the synergy between the qualitative and quantitative data, which enhances the overall clarity and flow of the argument.

**Criterion 6. The research meets ethical standards and is ethically sound**

The research received ethical approval from the University of Southern California Institutional Review Board and was deemed Exempt. The study followed ethical standards for research involving human participants, with focus group participants receiving an information sheet and survey participants receiving a short summary, where participation served as consent. The confidentiality of the qualitative transcripts was maintained, accessed only by the primary coder and principal investigator. The authors also declared that no competing interests exist.

**Criterion 7. The article adheres to reporting guidelines and community data availability standards**

The authors have demonstrated commitment to the PLOS ONE data policy. While full raw data cannot be shared due to confidentiality risks, the authors have confirmed with their IRB office that they will provide the aggregated minimal dataset upon acceptance of the manuscript. For the qualitative component, which requires transparent reporting, the authors have provided the complete codebook as supplementary information. This approach meets the journal's requirement that minimal data sufficient for replication must be made available.
