# Supplementary material for: US workforce gaps in emergency management: A mixed-methods approach of demographics, capacity, and community engagement
Source: PLoS One. 2026 Feb 17;21(2):e0342377. doi: 10.1371/journal.pone.0342377 (PMC12912579; doi:10.1371/journal.pone.0342377)
Supplement: Attachment — Submitted filename: Response to Reviewers_011926.docx [file pone.0342377.s010.docx]

Response to Reviewers

We thank the reviewers for the thoughtful and rigorous review. We have carefully addressed each point below and believe these revisions have strengthened the contribution of the manuscript.

The main points that still need to be comprehensively revised by the authors are confined to the Methods section and the Abstract, specifically:

**Comment:** Ensuring that the abstract follows the journal’s required structure for headings (introduction, methods, results, conclusions).

***Response: We have reviewed the journal’s required structure headings and have made the necessary revisions (changed “Purpose” to “Introduction”.)***

**Comment:** Providing a clearer and more explicit description of the methodology, including participant selection procedures, inclusion and exclusion criteria, sampling strategy, sample size justification with appropriate references, and data collection methods.

***Response: We have reorganized the Methods section and added explicit subheadings to clarify participant selection and data collection. We have also added a sentence about sample size justification.***

**Comment:** Clarifying the context of the sampled population (prehospital vs. intrahospital emergency settings), as this distinction is important for interpreting disaster management practices.

***Response: We have added a sentence that clarifies the context of the sample population.***
